# Impeding microbial biofilm formation and *Pseudomonas aeruginosa* virulence genes using biologically synthesized silver *Carthamus* nanoparticles

**DOI:** 10.1186/s12934-024-02508-9

**Published:** 2024-09-05

**Authors:** Sobhy S. Abdel-Fatah, Nasser H. Mohammad, Rana. Elshimy, Farag M. Mosallam

**Affiliations:** 1 Drug Radiation Research Department, Drug Microbiology Lab, Biotechnology Division, National Center for Radiation Research and Technology (NCRRT), Egyptian Atomic Energy Authority, Cairo, Egypt; 2 Radiation Microbiology Department, National Center for Radiation Research and Technology (NCRRT), Egyptian Atomic Energy Authority (EAEA), Cairo, Egypt; 3 Microbiology and Immunology, Egyptian Drug Authority, Cairo, Egypt; 4 Microbiology and immunology, Faculty of Pharmacy, AL-Aharm Canadian University (ACU), Giza, Egypt

**Keywords:** Ag-Carth-NPs, Radiation, Anti-biofilm, Kinetic growth, *P. aeruginosa* and anti-virulence genes

## Abstract

Long-term antibiotic treatment results in the increasing resistance of bacteria to antimicrobials drugs, so it is necessary to search for effective alternatives to prevent and treat pathogens that cause diseases. This study is aimed for biological synthesis of silver *Carthamus* nanoparticles (Ag-Carth-NPs) to combat microbial biofilm formation and *Pseudomonas aeruginosa* virulence genes. Ag-*Carth*-NPs are synthesized using *Carthamus tenuis* aqueous extract as environmentally friendly method has no harmful effect on environment. General factorial design is used to optimize Ag-*Carth*-NPs synthesis using three variables in three levels are *Carthamus* extract concentration, silver nitrate concentration and gamma radiation doses. Analysis of response data indicates gamma radiation has a significant effect on Ag-*Carth*-NPs production. Ag-Carth-NPs have sharp peak at λ max 425 nm, small and spherical particles with size 20.0 ± 1.22 nm, high stability up to 240 day with zeta potential around − 43 ± 0.12 mV, face centered cubic crystalline structure and FT-IR spectroscopy shows peak around 620 cm^−1^ that corresponding to AgNPs that stabilized by *C. tenuis* extract functional moiety. The antibacterial activity of Ag-*Carth*-NPs against pathogenic bacteria and fungi was determined using well diffusion method. The MIC values of Ag-*Carth*-NPs were (6.25, 6.25, 3.126, 25, 12.5, 12.5, 25 and 12.5 µg/ml), MBC values were (12.5, 12.5, 6.25, 50, 25, 25, 50 and 25 µg/ml) and biofilm inhibition% were (62.12, 68.25, 90.12, 69.51, 70.61, 71.12, 75.51 and 77.71%) against *Escherichia coli, Klebsiella pneumoniae, Pseudomonas aeruginosa*, *Bacillus subtilis, Staphylococcus aureus, Staphylococcus epidermidis*, *Candida tropicalis* and *Candida albicans* respectively. Ag-*Carth*-NPs has bactericidal efficacy and significantly reduced the swarming, swimming motility, pyocyanin and protease production of *P. aeruginosa*. Furthermore, *P. aeruginosa ToxA* gene expression was significantly down regulated by 81.5%, while *exoU* reduced by 78.1%, where *lasR* gene expression reduction was 68%, while the reduction in *exoU* was 66% and 60.1% decrease in *lasB* gene expression after treatment with Ag-*Carth*-NPs. This activity is attributed to effect of Ag-Carth-NPs on cell membrane integrity, down regulation of virulence gene expression, and induction of general and oxidative stress in *P. aeruginosa.* Ag-Carth-NPs have no significant cytotoxic effects on normal human cell (Hfb4) but have IC_50_ at 5.6µg/mL against of HepG-2 cells. Limitations of the study include studies with low risks of silver nanoparticles for in vitro antimicrobial effects and its toxicity.

**Supplementary Information:**

The online version contains supplementary material available at 10.1186/s12934-024-02508-9.

## Introduction

Nanotechnology has emerged as one of the most promising technologies to overcome the crisis of drug resistance microbes [1–4]. Chemical vapor deposition, microwave, laser ablation, ultrasonic radiation, and electrochemical methods are a few frequently used for metallic nanoparticles synthesis, because these methods are characterized by high production costs and the use of hazardous and toxic compounds, which may have detrimental impacts on human health and the environment [5]. Green synthesis in which mostly uses plant extracts, is an environmentally acceptable method to produce metallic nanoparticles without having to deal with hazardous chemicals, because compounds derived from plant extracts (flavonoids, polyphenols, proteins, sugars, and saponins) act as stabilizing and reducing agents metals instead hazardous chemicals [6].The biological synthesis of nanoparticles using plants and plant extracts appears to be an attractive alternative to conventional chemical synthesis [7–9]. Recently, synthesizing metal nanoparticles using plants has been extensively studied and has been recognized as a green and efficient method [10, 11]. The biological synthesis of metal nanoparticles has low toxicity when compared with other methods [12]. They are used natural extracts for example, as tomato, paprika, or marigold extracts, fenugreek, lupin, and other plants for biological synthesis of metals nanoparticles [13–15]. Use of *Gymnanthemum amygdalinum* extracts for Se-NPs biosynthesis [16]. Synthesis of copper nanoparticles Using *Krameria* sp. Root extract [17]. Leaf extracts of *Clerodendrum Inerme* were used for the generation of silver nanoparticles [18].

There are about 47 species in the genus *Carthamus* (family Asteraceae), 15 of which are found in Western Asia and the Middle East region, *Carthamus tenuis *is one of them [19]. While several of these species were explored for their potential medical uses, it’s odd that not much research was done on *C. tenuis*, despite the fact that it’s widely distributed throughout the Middle East [20]. *C. tenuis*leaves and stems aqueous extract it has not been used before in the formation of nanoparticles and is neglected in use in the Middle East. Most previous researches are focused on *Carthamus tinctorius* no *Carthamus tenuis*. Additionally, they show that the plant is traditionally used in Egypt as an aphrodisiac, to promote fertility, to prevent abortions and as antimicrobial herbal medicine[21]. The crop holds significant industrial value as a result of its ability to extract commercial oil. Moreover, the substances found in its petals serve as natural food coloring [22]. Nevertheless, after the seed is extracted, over 80% of this product (leaf or stem) is regarded as an agricultural waste [23]. Flavonoids, quinochalcones, polyacetylenes, alkaloids, fatty acids, steroids, lignans, proteins, and polysaccharides are among the chemical families that have been identified from safflower; quinochalcones and flavonoids are the primary active chemicals [24, 25], that play important role in reduction and stabilization of nanoparticles. Plant extracts have been shown to possess antibacterial properties due to the presence of phenolic chemicals [3, 26]. Safflower’s antibacterial activity is probably caused by polyphenols rupturing membranes and allowing biological components to escape. Thus, substances that disrupt membranes cause cellular contents to leak out and obstruct metabolic enzymes, which inactivates bacteria[27].

Recently, as an alternative to antibiotics, the use of different nanoparticles (NPs) to treat bacterial infections [28, 29] and fungal infection has been increasing in popularity [30]. A significant advancement in nanotechnology is the use of silver nanoparticles compared to other metals, because of their great stability, low chemical reactivity and have special physicochemical features, they have drawn a lot of interest in biological applications [31]. The high surface area and distinct chemical and physical properties of silver nanoparticles have made them effective antimicrobial agents against multidrug resistant microbes [9]. Silver nanoparticles remarkable antibacterial properties make it an essential tool for tissue regeneration, cancer treatment, wound healing, and infection prevention[32]. Furthermore, AgNPs exhibit significant potential as adaptable drug carriers for targeted treatments and as contrast agents for cutting-edge medical imaging methods [33]. Silver nanoparticles can kill organisms by causing breakdown of the cytoplasmic membrane and cell wall, then liberating silver ions (Ag +) to adhere to or go through the membrane, causing ribosome denaturation and stopping protein production [34].According to their earlier research, biofilm formation in *Escherichia coli*, *Pseudomonas aeruginosa*, and *Klebsiella pneumoniae* was inhibited within 24 h by increasing the concentration of silver nanoparticles from 12.5 to 100 μg/ml [35]*.* Silver Nano that applied to textile materials for wound dressing can prevent bacterial adhesion and biofilm formation [36].

Bacterial biofilms are bacterial communities embedded in a selfproduced polymeric matrix (extracellular polymeric substance) that adheres to surfaces and their interface, this biofilm shields the bacterium from antimicrobial agents and host immune responses [37].Biofilm forming organisms have a natural resistance to antibiotics, disinfectants, and germicides [38, 39]. The biofilm bacteria can withstand higher antibiotic concentrations than bacteria in suspension makes it more difficult to eradicate a persistent illness linked to biofilm formation [40]. The production of several virulence factors further aids pathogenicity and infection by biofilm-forming bacterial pathogens [41]. The bacterial infections that produce biofilms are responsible for causing mutant cells to become more resistant to antibiotics [42]. It is now widely known that biofilms are extremely difficult to remove and frequently resistant to systemic antibiotic therapy [43]. *P. aeruginosa* is a gram negative bacterium widely distributed in the environment, usually inhabiting soil, water, plants, and humans [44]. *P. aeruginosa* infects individuals suffering from immunodeficiency, cystic fibrosis, burn wounds, cancer, chronic obstructive pulmonary disease (COPD), and severe infections that need to be ventilated, such COVID-19 [45]. In all biological domains, *P. aeruginosa* is also a widely used model bacteria[46]. *P. aeruginosa* virulence factors that are related to disease development in healthy organisms and resistance to antibiotics [47]. The use of nanoparticles to inhibit *P. aeruginosa* virulence factors is a promising strategy [48]. Biologically synthesized silver nanoparticles are reported as anti-QS and antibiofilm drugs against *P. aeruginosa* infections[49]. Previous study show a significantly decreased the expression of quorum sensing regulatory genes of *P. aeruginosa; lasR, lasI, pqsA, fliC, exoS and pslA* with Ag-MET-NEs [50]. Ag-nanoparticles can inhibit virulence factors of microbes, this action mechanism is related to their effect on the integrity of cell membranes, the down regulation of the expression of virulence genes, and the creation of oxidative and general stress in microorganisms[51]. Novelty of the study is Ag-Carth-NPs using *C. tenuis* aqueous extract, where *C. tenuis* it has not been used before in the formation of nanoparticles and is neglected in use in the Middle East.

*The current study aimed* to biological synthesis of silver *Carthamus* nanoparticles (Ag-Carth-NPs) to combat pathogenic microbe’s biofilm formation and *P. aeruginosa* virulence genes.

## Materials and methods

### Chemicals

The ingredients of the media utilized in the microbiological examination were obtained from Oxoid, and silver nitrate, Clindamycin, Nystatin, Crystal Violet, ethanol was obtained from Sigma-Aldrich.

### Preparation of safflower (*Carthamus tenuis*) aqueous extract

#### Plant sample

Fresh samples of *Carthamus tenuis were* collected locally from Abu El Matamir, Al Buhayrah, Egypt, 2023. The *C. tenuis* waste used in the present investigation consisted of a mixture of stem and leaf obtained after the seed collection process. The sample was collected 150 days after sowing***.*** The plant samples leafs and stems were cleaned using sterile distilled water and then dried at room temperature (25 ℃ for 1 weeks), after drying the leaf and stem samples was ground into powder using a blender. The dried plant materials were stored in a dark area until needed.

#### Extraction of bioactive compounds

Extraction of *Carthamus tenuis* was performed according to the modified method described by [52]. Briefly, 5.0 g of fine safflower powder was immersed in 100 ml of sterile distilled water for 3 h on magnetic stirrer at 80 ℃. Finally, it was centrifuging the macerate at 4000 rpm and 5 ℃ using a cooling centrifuge, the supernatants were was recovered and filtered on Whatman #4 paper, and then dried at room temperature (25 ℃) and stored at 5 ℃ until used for nanoparticles synthesis.

### Preparation of Silver- *Carthamus* nanoparticles

#### Optimization of silver- *Carthamus* nanoparticles (Ag-Carth-NPs) synthesis

A general factorial design was used to compare the influence of different parameters on the synthesis of silver nanoparticles [15]. The statistical software package used was Design-Expert 7.0 (Stat Ease Inc., Minneapolis, U.S.A) to determine the factors that have positive and significant effects on the process. Certain factors were chosen as they have the most meaningful impact on AgNPs synthesis. The influences of the three variables in three levels are *Carthamus* extract (1, 2, and 3 × 10^2 ^µg/ml disolved in DMSO), silver nitrate concentration (0.5, 1and 1.5 × 10^2 ^µg/ml disolved in sterile deionized water), and gamma radiation doses (0, 1, and 5 kGy). All samples was mixed by v/v using magnetic stirrer at 25 ℃. and then exposed to gamma radiation according to Table 1. Responses as optical density (OD) was meaured after radiation using UV-visible spectroscopy at wavlentgh 425 nm to estimated how the main parameter affects the synthesis of Ag-Carth-NPs after 24 h of reaction time.

**Table 1:** Experimental factors that have an effect on Ag-Carth-NPs production

Run no	Factors			Response
	Ag-nitrate (µg/ml) × 10^2^	Radiation dose	*Carthamus* dry extract (µg/ml) × 10^2^	Optical density (OD) of Ag-Carth-NPs at 425nm
1	0.5	1	2	1.3 ± 0.01
2	1.5	1	1	1.03 ± 0.05
3	1	5	1	2 ± .012
4	1.5	5	2	2.4 ± 0.031
5	1	5	3	2.1 ± 0.041
6	1.5	1	3	1 ± 0.41
**7**	**1**	**5**	**2**	**4.42 ± 0.21**
8	1.5	5	1	2.6 ± 071
9	1	1	2	3.12 ± 0.031
10	1.5	0	3	1.23 ± 0.041
11	1	1	1	2.3 ± 0.071
12	0.5	1	3	1.9 ± 0.081
13	0.5	0	3	1.032 ± .057
14	1.5	0	1	1.16 ± 0.019
15	1.5	0	2	1.6 ± 0.018
16	0.5	0	1	1.32 ± 0.061
17	1	0	2	1.7 ± 0.026
18	0.5	5	1	1.84 ± 0.061
19	0.5	5	3	2.3 ± 0.01
20	1	0	1	1.02 ± 0.038
21	0.5	1	1	2.15 ± 0.031
22	0.5	0	2	1.3 ± 0.003
23	1.5	5	3	1.95 ± 0.019
24	1	1	3	2.054 ± 0.019
25	1.5	1	2	2.15 ± 0.017
26	1	0	3	1.2 ± 0.019
27	0.5	5	2	1.94 ± 0.12

Analysis of variance table^a^					
Source	Sum of squares	df	Mean square	F Value	**p-value Prob > F**
Model	9.03	6	1.50	5.20	**0.0023**
A-Ag	1.72	2	0.86	2.96	**0.0746**
**B Radiation**	5.56	2	2.78	9.60	**0.0012**
C-Extract	1.76	2	0.88	3.03	**0.0707**
Residual	5.79	20	0.29		
Cor Total	14.82	26			

The statistical software package used was Design-Expert 7.0 (Stat Ease Inc., Minneapolis, U.S.A). Where the comparison tests were performed for values of similar responses (p < 0.05)

Bold values are refer to P values

^a^Significant**: **The Model F-value of 5.20 implies the model is significant. There is only a 0.23% chance that a “Model F-Value” this large could occur due to noise. Values of “Prob > F” less than 0.0500 indicate model terms are significant. In this case B is significant model terms

#### Validation of Ag-Carth-NPs and stability

The size and morphology of the synthesized Ag-Carth-NPs were characterized using the following techniques. Transmission electron microscope (TEM) (TEM-JEOL electron microscope JEM-100 CX) operating at 80 kV accelerating energy is great for characterizing the size and shape of nanoparticles [53]. Particle size, particle size distribution and Zeta potential was determined by Dynamic light scattering (DLS) Zetasizer Technique (PSS-NICOMP 380-ZLS, USA) where 250 μl of suspension transferred to a disposable small volume cuvette. While the Fourier transforms infrared spectroscopy (FT-IR) was employed to assess the function moiety [54], where the samples were recorded in KBr pellets using an FT-IR (JASCO FT-IR -3600). Using X-ray diffraction analysis, the crystalline structure of the produced metallic nanoparticles [55], was established. Cu-Ka target and nickel filter were used in the study of extended X-ray diffraction models (Shimadzu apparatus -Shimadzu Scientific Instruments; SSI, Japan). Operating at 50.0 mA and 40.0 kV, with a flow rate of 2/min, and a 2 h value between 20 and 100, a Cu anode is used. The intensity of the diffracted X-rays is determined based on the diffracted angle 2 h.

The stability of Ag-Carth-NPs was estimated at different storage time ranged from 1 to 240 day at room temperature (25 ℃ and neutral pH (7.0) using by UV-vis spectroscopy [56, 57]. Further, the zeta potential was measured to assess the stability and solubility of Ag-Carth-NPs in aqueous solution.

### Antimicrobial assays

#### Microorganism

Microbial strains: In this study standard strain were kindly provided from culture collection of Drug Microbiology Lab, Drug Radiation Research Department, Biotechnology Division, National Center for Radiation Research and Technology (NCRRT), Egyptian Atomic Energy Authority. In addition to clinical isolates of *P. aeruginosa* were used and selected after screening of more than 50 *P. aeruginosa* clinical isolate. The selection of *P. aeruginosa* clinical isolates based on higher resistant to free silver nitrate and *Carthamus* extract solution. Preparation and sterilizing the agar plates was done according to previous described study [58], with slightly significant modification (Supplementary data).

A colony of the required strain was picked from the stock plates and transferred to 10mL of LB medium to prepare the overnight bacterial cultures (37 ℃, 200 rpm, using an orbital shaker incubator). The overnight cultures were refreshed by adding fresh LB medium and incubated at 37 ℃ for approximately 1 h. We aimed to reach the appropriate OD 600 corresponding to the known concentration of bacteria expressed as CFU per ml. Each bacterial culture was diluted in sterile 0.9% NaCl to an initial concentration of about 1 × 10^6^ cells per mL.

#### Inhibition zone diameter

The antimicrobial activity of Ag-Carth-NPs was determined by using the agar diffusion method [59, 60] against gram positive bacteria *Staphylococcus aureus* ATCC 25923*, Staphylococcus epidermidis* ATTC 12228 and *Bacillus subtilis* ATCC 6633 and gram negative bacteria like *Escherichia coli* RCMB 0020B01*, Klebsiella pneumoniae* ATTC 13883*, P. aeruginosa* clinical isolate; additionally, antifungal towards *Candida tropicalis RCMB001Y004* and *Candida albicans* ATCC 10231.

The agar plate surface is inoculated by spreading a volume of the microbial inoculum (1 × 10^6^ cells per ml) over the entire agar surface. Then, a well (with a diameter of 6 mm) is made aseptically using a sterile corkborer. The well was diffused with 50 µl of each tested sample: Ag-Carth-NPs at 100 µg/ml. *Carthamus* extract (2 × 10^2 ^µg/ml) and silver nitrate (1 × 10^2 ^µg/ml) was used as negative control and Clindamycin 2 μg/ml and Nystatin 100 μg/ml as positive control, then incubated at 37 ℃ for 24 h [61, 62]. The measurement of inhibition was carried out by observing the emergence of a clear zone.

#### Minimum inhibitory concentration (MIC) determination and minimum bactericidal concentration (MBC)

The MIC was determined by well diffusion method based on the guidelines of the Clinical Laboratory Standard Institute (CLSI) [63]. The MIC of Ag-Carth-NPs was identified to determine the lowest concentration that inhibits the visible growth of the test organisms. Different concentrations of Ag-Carth-NPs (100, 50, 25, 12.5, 6.25, 3.125, and 1.625 µg/ml) were used. The procedure was repeated three times and the mean value was taken into consideration. In these experiments, a positive control (nutrient plus microorganism), and negative control one (the nutrient only) had been used. The data within the samples are analyzed using one way analysis of variance (ANOVA).

After the MIC determination of the Ag-Carth-NPs, aliquots of 10 µl samples from all the tubes which showed no visible bacterial growth were seeded on Brain Heart Infusion Agar (BHI agar) plates and incubated for 24 h at 37 ℃. When 99.9% of the bacterial population is killed at the lowest concentration of an antimicrobial agent, it is termed as the minimum bactericidal concentration (MBC) endpoint [64]. This was done by observing pre and post-incubated agar plates for the presence or absence of bacterial colonies. The procedure was repeated three times and the mean value was taken into consideration. The data within the samples are analyzed using one way analysis of variance (ANOVA).

#### Anti-biofilm activity

The biofilm qualitative and quantitative formation analysis was performed in the absence and presence of Ag-Carth-NPs against all tested organisms. A semi-qualitative detection of biofilm formation was determined according to Elbasuney, [65]. Fifty microliters of the chosen microbes overnight culture in LB, the broth turbidity was adjusted at 0.5McFarland Standards (5 × 10^5^ CFU/ml). The microbial suspension was put to the tubes contain two milliliters of sterilized LB broth, and these tubes were kept at 37 ℃ for 1 day after addition of Ag-Carth-NPs at sub-MIC (0.25MIC, 0.5MIC and 0.75MIC) to each separate tubes. The experiment also included tubes containing media alone represented negative controls and tube contain media plus tested organisms in absence of Ag-Carth-NPs represented as positive control. Following incubation, the broth culture was poured out and washed with three PBS (PBS; pH 7.0). Next, the bacterial and yeast cells biofilms that adhered to the tube walls were fixed using sodium acetate (3.5%) for approximately 20 min. For thirty minutes, the insides of the tubes were stained with 10 mL 0.1% crystal violet dye. While the excess dye was decanted and washed off with deionized water gently, the tubes were dried and the biofilm formation ability was determined by observing a thin layer of blue film on the walls of tubes.

The microtiter plate technique was utilized for quantitative estimation of biofilm formation [66]. Using 96-well microtiter plates, the assay involved inoculating each well with 100 μl of LB broth, 10 μl of culture cultured overnight and diluted further to a final concentration of 5 × 10^5^ CFU/ml, and 10 μl of Ag-Carth-NPs at sub-MIC (0.25MIC, 0.5MIC and 0.75MIC) was measured against the chosen microbes. After being incubated at 37 ℃ for 24 h, the contents of the wells was carefully removed and three times cleaned with PBS (PBS; pH 7.0) to get rid of any bacteria. After that, biofilms were fixed with sodium acetate sodium acetate (3.5%) and stained for ten minutes with 50 μl of 0.1% crystal violet dye. After being dyed and affixed to wells, the cells were dried and cleaned with distilled water. 200 μl of 95% ethanol was added in each well to elute the attached cells, and absorbance was measured at 600 nm on ELISA reader in order to quantify cells capable of forming biofilms. Negative and positive controls were also used in the assay.

The inhibition percentage was calculated using equation (1)

1 $$Percentage \,of \,biofilm \,\,inhibition \,(\%) = 1- \left(OD \,of \,\,treated \,with \,Ag-Carth-NPs/OD \,of \,untreated \,control\right) \,x \,100$$

#### Time-Kill curve assay

Time-kill assay was done in MHB medium as described by Loo et al. [9]. The inoculums were adjusted to 10^6^ CFU/millimeter. The Ag-Carth-NPs solution was diluted with MHB media containing microbial inoculums to obtain the final concentration of 0 MIC, 0.5 MIC, 1 MIC, 2MIC and 4MIC, for each type of microbes in the total final volume of 1 ml. The cultures were then incubated at 37 ℃ with 140 rpm agitation. The cultures (100 µl) were spread on MHA plates at time 0, 1, 2, 4 and 8 h. The experiment was carried out in triplicate. The number of colonies on the MHA plates was quantified in CFU/mL after incubation at 37 ℃ for 24 h and then, their growth rate was identified through reading OD600 using fixed UV- spectroscopy.

Statistical Analysis- One way ANOVA: The results represent the mean±SD from at least three independent experiments. One-way ANOVA with Tuckey post-hox test, using Graph 10 software (*P<0.05; **P<0.01 and ***P<0.001) was performed (*P<0.05; **P<0.01 and ***P<0.001) compare to the control group.

#### Motility of *P. aeruginosa* assay

*Swarming motility assay* In order to test the capacity of the Ag-Carth-NPs to block the swarming motility of *P. aeruginosa* clinical isolate was performed as discussed earlier [67]. A clinical isolate of *P. aeruginosa* was grown in Luria Bertani broth (LB) for 24 h then bacterial suspension was adjusted to reach an OD600=1. The swarming medium composed of LB along with 0.5% (w/v) casamino acids and 0.4% (w/v) Bacto agar. Before agar solidification, 0.5MIC concentration of Ag-Carth-NPs was added, and then 2.5 µl fresh bacterial culture was placed on the surface of the medium, followed by incubation at 35 ℃ for 24 h. and bacterial swarming zone was then measured [68]. All motility experiments were performed in triplicate. All motility experiments were performed in triplicate. Tubes contain media plus *P. aeruginosa* in absence of Ag-Carth-NPs represented as positive control.

*Swimming assay* Ag-Carth-NPs were tested for their impact on *P. aeruginosa* using a plate-based swimming assay. To put it briefly, the petri dishes were filled with Luria broth medium that included 0.3% agar and 0.5MICs Ag-Carth-NPs. Next, bacterial cells were added to the plate’s agar layer using sterile yellow pipette tips (not to the base of the Petri plate). After a 24-hour incubation period, the swimming phenotype's radial growth was measured on the plates [69]. Tubes contain media plus *P. aeruginosa* in absence of Ag-Carth-NPs represented as positive control.

#### Pyocyanin level

In order to determine how Ag-Carth-NPs affected *P. aeruginosas* capability to produce pyocyanin, bacterial cultures in an LB medium containing 0.5MIC Ag-Carth-NPs were created in tubes. The tubes were shaken at 80 rpm for 48 h at 37 ℃. Following centrifugation of the cultures, samples of the cell-free supernatant (CFS) were taken. After that, 4 ml of the CFS samples were mixed with 2 ml of chloroform, vortexed, and centrifuged for 15 mins. Then, the chloroform layer was transferred to a fresh tube and mixed with 1 ml of 0.2 M HCl. After centrifugation again, the top layer was removed and OD at 520 nm was measured [70]. Tubes contain media plus *P. aeruginosa* in absence of Ag-Carth-NPs represented as positive control.

#### Total proteases inhibition assay

Using the modified skimmed milk broth method, the effect of Ag-Carth-NPs on inhibition of total proteases by *P. aeruginosa* isolates was investigated. To acquire the supernatants, *P. aeruginosa* overnight cultures in MHB with and without 0.5MIC of the Ag-Carth-NPs were centrifuged. 500 μl aliquots of bacterial supernatants were cultured for 1 h at 37°C with 1 ml of 1.25 percent skim milk. Using a Biotek spectrofuorometer (USA), the drop in optical density of skimmed milk was measured at 600 nm and was thought to be estimate for proteolytic activity [50]. Tubes contain media plus *P. aeruginosa* in absence of Ag-Carth-NPs represented as positive control.

#### Gene expression

Effect of Ag-Carth-NPs on relative genes expression *P. aeruginosa* clinical isolate were tested for virulence genes using real-time reverse transcriptase-polymerase chain reaction (rt-PCR) according to the following steps

*Bacterial DNA extraction* DNA extraction from samples was performed using the QIAamp DNA Mini kit (Qiagen, Germany, GmbH) as previously described [71] with modifications from the manufacturer’s recommendations. Oligonucleotide Primer; Primers used were supplied from Metabion (Germany) are listed with specific references [72–75] in Table 1s. PCR reaction and Analysis of the PCR Products were performed in an applied biosystem 2720 thermal cycler as published before [76].

##### Bacterial RNA extraction and quantitative real-time PCR (qRT-PCR)

Total RNA was isolated from clinical isolate stain of *P. aeruginosa* cells cultured in the presence or absence of Ag-Carth-NPs (0.5MIC) in order to analyze the expression genes. The RNeasy Mini kit (Qiagen, Germany, GmbH) and the TRIzol reagent (Invitrogen, Waltham, MA, USA) were used for the extraction process. To eradicate DNA contamination, the RQ1-DNAse kit (Promega, USA) was utilized. The A260/A280 ratio calculation and agarose gel electrophoresis were used to confirm the quality of the extracted RNA. Primer-BLAST and BioEdit Sequence Alignment Editor were used to generate and analyze primers after gene sequences were taken from Gen Bank. The primers pair’s sequences for individual genes are presented with references [72, 75] in Table 2s. The QuantiNova SYBR Green RT-PCR kit (QIAGEN, Germantown, MA, EUA) was used to perform RT-qPCR in a final volume of 20 µl. The kit contained 10 µl of SYBR Green RT-PCR Master Mix, 0.2 µl of RT mix, 1 µl (20 µM) of each primer, 5 µl of bacterial RNA (50 ng/µl), and 2.8 µl of RNase-free water. Using the Rotor-Gene Q 2plex (QIAGEN, Germantown, MA, EUA) for the reaction, the following procedures were followed: 10 min of reverse transcription at 50 ℃, 2 min of initial denaturation at 95 ℃, 40 cycles of 95 ℃ for 5 s, and 10 s of hybridization and extension at 60 ℃. The changes in the expression level of target gene were analyzed by using the method adopted by Livak and Schmittgen [77].

#### Evaluation of cytotoxic effects

The cytotoxic action of Ag-Carth-NPs on the Hfb4 cells (normal skin cell lines) and HepG-2 cells (human Hepatocellular carcinoma) was determined as previously described by [54, 78];. A volume of 100 µl/well of Ag-Carth-NPs at concentrations 100, 50, 25, 12.5, 6.25, 3.125 and 1.56 µg/ml was injected into the 96-well microliter plate containing 1 × 10^6^ HepG-2 cells or and Vero cells. The samples were incubated for 4 h at 37 ℃, 5% CO_2_. Controls are wells were left without Ag-Carth-NPs. The absorbance at λ=570 nm was measured using a plate reader. The relation between surviving cells and Ag-Carth-NPs concentration is plotted to get the survival curve of each tumor cell line after treatment with the specified compound The Cytotoxic concentration (CC_50_), the concentration required to cause toxic effects in 50% of intact cells, was estimated from graphic plots of the dose response curve for each conc. using Graphpad Prism software (San Diego, CA. USA).

### Statistical analysis

The results represent the mean±SD from at least three independent experiments. One-way ANOVA with Tuckey post-hox test, using Graph 10 software (*P<0.05; **P<0.01 and ***P<0.001) was performed (*P<0.05; **P<0.01 and ***P<0.001) compare to the control group.

## Results and discussion

### Optimization of silver- *Carthamus* nanoparticles synthesis

The experimental factorial design was used in this study for screening factors that significantly effect on the final Ag-Carth-NPs production. Responses optical density (O.D) estimated how the main parameter effects on the Ag-Carth-NPs production (Table 1) where, OD values is was triplicate measured at fixed wavelength 425 nm using UV-visible spectroscopy. All parameters, such as concentration of *Carthamus* extract (1, 2, and 3 × 10^2 ^µg/ml), silver nitrate concentration (0.5, 1 and 1.5 × 10^2 ^µg/ml), and gamma radiation doses (0, 1, and 5kGy) were maintained constantly.

The results showed that, trial no (7) has high peak optical intensity (O.D a.u) about 4.42 ± 0.21 in competing with other trials that show optical density at range from 1.02±0.038 to 3.12±0.031 (Table 1); where high value of OD indicates higher yields of NPs [79]. The variation in the OD value coincides with nanoparticles production amount [80], trial with high OD indicate high yield of NPs than trial with low OD [81]. Factorial design indicates that, the maximum yield of Ag-Carth-NPs (OD = 4.42) was achieved with run corresponds to an actual 1 × 10^2 ^µg/ml Ag-nitrate, 5kGy and 2 × 10^2 ^µg/ml *Carthamus* dry extract. Analysis of response data show Values of “Prob > F” less than 0.0500 indicate radiation is significant model terms. The peak optical density increased proportionately, that is mainly due to the high production of nanoparticles [82, 83]. Optimization of lupin-Se-NPs production using general factorial design was previously discussed [14]. Gamma ray improve metallic nanoparticles synthesis in presence of plant extract [84], this attributed to potent reducing free electron generated from radiation that assist reduction of ions to metallic nanoparticles [85, 86].

**Fig. 1:**
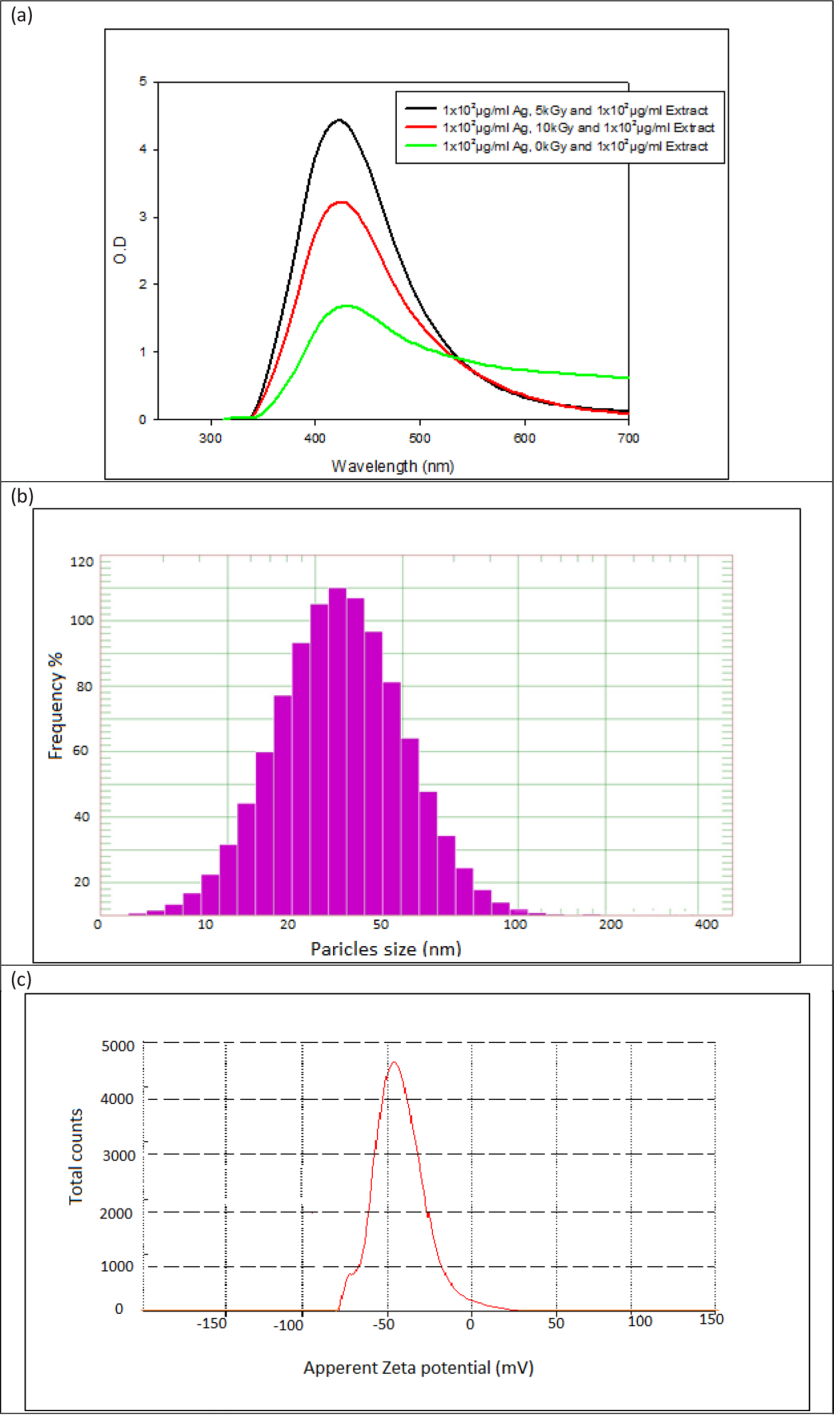

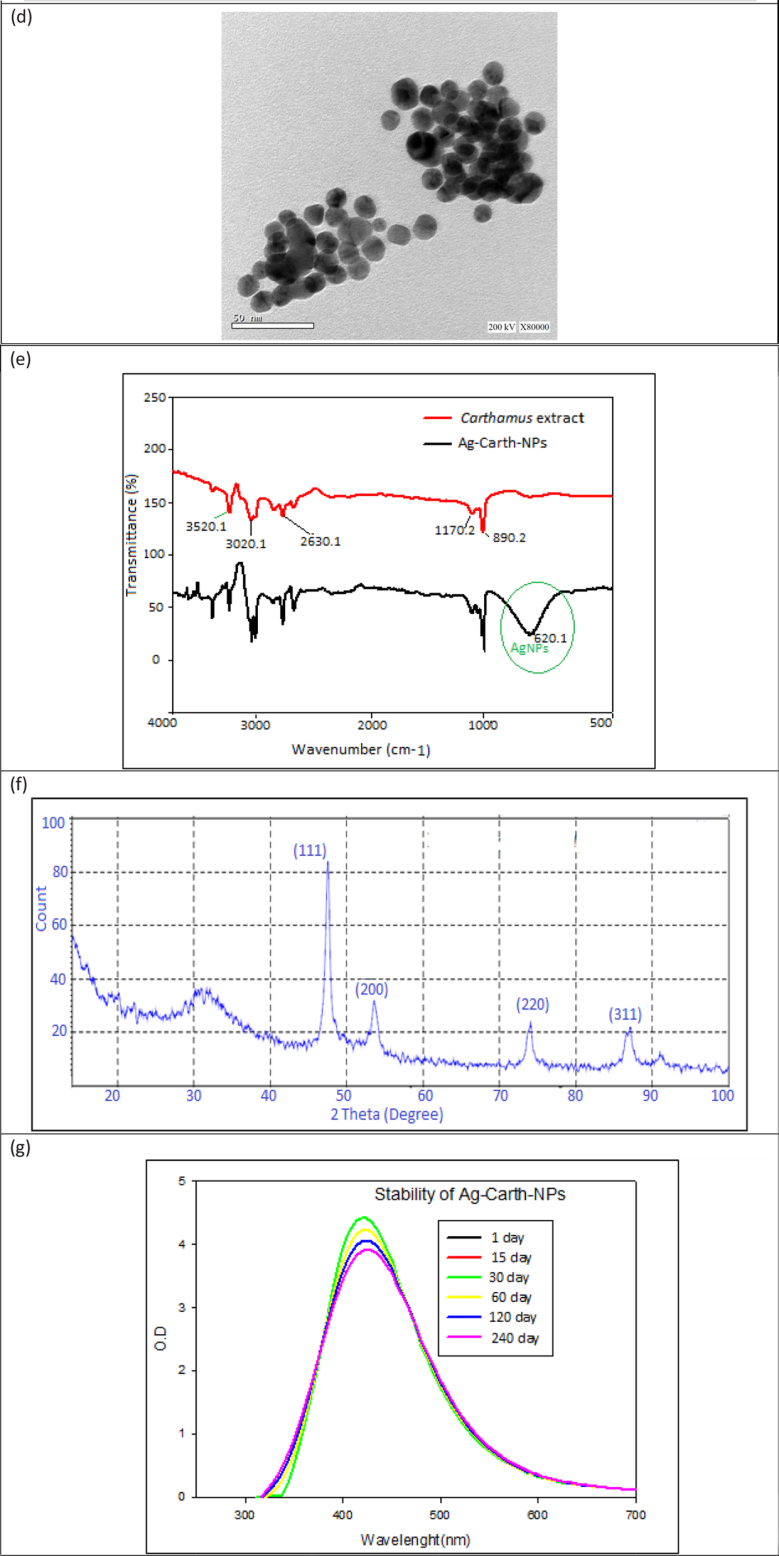
**a** UV–visible scanning of Ag-Carth-NPs, **b** DLS diagram of Ag-Carth-NPs, **c** diagram of Ag-Carth-NPs Zeta potential, **d** TEM image of diagram of Ag-Carth-NPs, **e** FT-IR spectrum of Ag-Carth-NPs, **f** XRD pattern of Ag-Carth-NPs and **g** UV–visible scanning of Ag-Carth-NPs stability over time

The conditions 1 × 10^2 ^µg/ml Ag-nitrate, 5kGy and 2 × 10^2 ^µg/ml *Carthamus* dry extract, with change in gamma radiation dose by increase or decrease the dose show negative effect on Ag-Carth-NPs production (Figure 1a). This attributed to increase in random movement of particles in Ag-Carth-NPs suspension, that associate with increase of gamma doses leading to Ag-Carth-NPs aggregation and precipitation [86], or decrease in Ag-Carth-NPs production that associated with decrease in gamma radiation due to low level of free electron production that responsible for silver reduction [85].

### Ag-Carth-NPs validation and stability

UV-Vis spectrophotometry: For the first validation of metallic nanoparticle production, UV Vis spectrophotometry is an essential tool [87]. The UV-Vis spectrum of Ag-*Carth*-NPs (Figure 1a) showed the highest absorption peaks at 425nm in solutions, which indicate the localized surface Plasmon resonance (LSPR) characteristics of silver nanoparticles [88]. Smaller nanoparticles absorb light primarily and have peaks that are near 400 nm, whereas larger particles show more scattering and have peaks that broaden and shift toward longer wavelengths (a process called red-shifting) [89]. Figure 1a show that, Ag-*Carth*-NPs UV-Vis spectrum at 0kGy, 10kGy at 1 × 10^2^µg/ml Ag-nitrate and 2 × 10^2 ^µg/ml *Carthamus* dry extract has the same peak wavelength 425 nm with decrease in optical density (1.7±0.031 and 3.23±0.161 respectively) compering with 5kGy (4.42±0.21), this indicates low Ag-*Carth*-NPs production with decrease or increase of gamma radiation dose. Where increase in optical density corresponds with a dosage of gamma rise from 0 to 5kGy and then decreased with 10kGy. an increase in peak intensity, indicating a decrease in particle size and an increase in NPs production[85]. The results from UV-Vis spectrum of Ag-*Carth*-NPs and general factorial design optical density response confirm gamma radiation has significant effect on Ag-*Carth*-NPs production yield and the optimum dose is 5kGy.

The particle size distribution and zeta potential of Ag-Carth-NPs in water dispersion was measured by DLS technique [90]. Ag-Carth-NPs size distribution was found at range 32.00 nm (Figure 1b). Dynamic Light Scattering (DLS) estimates the hydrodynamic diameter of dissolved nanoparticles and gives insights on their aggregated state [91]. Size dispersed particle averages of DLS values are slightly greater than TEM values. Because, DLS you measure hydration sphere diameter where there will be solvent molecules associated with your particle [92].

Zeta potential is it an important tool for understanding the state of the nanoparticle surface and predicting the long term stability of a colloidal dispersion [93]. Figure 1c show Zeta potential of Ag-Carth-NPs at range about − 43±0.12 mV. The negative zeta potential indicated the stability of the nanoparticles and prevent them from agglomeration[94]. According to previously published research, nanoparticles that have a zeta potential less than – 30 mV are thought to be highly stable in the dispersion medium [95].

Transmission electron microscopy (TEM) it’s a quantitative technique to determine the particle size, shape and distribution [96]. The TEM images clearly demonstrated the Ag-Carth-NPs has spherical isotropic shape and anisotropic particles, with average particle sizes ranging 20 ± 1.22 nm (Figure 1d). TEM results are based on a number analysis of a dry particle and the values tend to be smaller than DLS results [97], this because TEM reveals the structure of molecule from interior and gives thought approximately molecule diameter and framework structure in solid case [98]. The presence of *Carthamus* extract act as capping and stabilizing agents that prevents the aggregation and agglomeration of generated Ag-Carth-NPs.

FTIR spectrum is used to identify the functional groups used in capping of NPs [99]. FT-IR spectrum (Figure 1e) evidences the presence of different functional groups of biomolecules participated in stabilization NPs [100]. Accordingly, the FTIR spectrum of the *Carthamus* extract exhibited several peaks around 3520, 3020, 2630, 1170 and 890 cm−1 that indicate presence of (O-H), (N-H), (C-O) and (RCOO) function moiety. These characteristic bands predict that in the extract there are structures as proteins, polysaccharides/sugars and phenolic compounds, mainly flavonoids[101]. Results of FT-IR Ag-Carth-NPs analysis shows absorption peak at 620 cm^-1^ corresponding to presence of AgNPs [102], that absent from *Carthamus* extract. The FTIR results we can conclude that some of the biological compounds from *Carthamus* extract formed a strong capping agent on the AgNPs for their stabilization.

Figure 1f illustrates the XRD pattern for Ag-Carth-NPs, which showed several peaks related to Ag-Carth-NPs. Within 2 h (degree), the diffraction characteristics are 37.04, 43.11, 65.31, and 76.51, respectively. These peaks correspond to the Bragg’s reflections (111), (200), (220), and (311) planes. This suggests that silver nanoparticles have a face-centered cubic (fcc) crystalline structure [59]. The XRD pattern shows amorphous regions before 30.0 degree of 2 theta, its due to presence of biological extract [103].

The stability of Ag-Carth-NPs over a range of time was estimate firstly using UV-vis spectroscopy (Figure 1g), the intensity and sharpness of peak and location has no changed after 30 day of storage this indicate highest stability and no changes in size or distribution of nanoparticles [104, 105]. Peak intensity of Ag-Carth-NPs are slightly decreased with time increased after 60 day up to 240 day with no changes in wavelength location or sharpness, this indicate slightly aggregation of NPs [106].The stability of Ag-Carth-NPs is also estimated by DLS and zeta potential (Table 2), that show Ag-Carth-NPs has DLS size distribution at range from 32.00 ± 1.55 nm to 37.51 ± 1.71 nm at time storage ranged from 1 to 240 day with zeta potential ranged from – 43 ± 0.12 to -47±0.81 mV and Polydispersity index value at range from 0.211 ± 0.05 to 0.321 ± 0.09. These results confirm high stability of Ag-Carth-NPs over longer time with no significant changes in size with time storage.

**Table 2:** The stability of Ag-Carth-NPs at different time

Time (day)	Wavelength (nm)	Absorbance O.D	DLS (nm)	Zeta potential (mV)	Polydispersity index (PDI)
1	425	4.42	32.00 ± 1.55	− 43 ± 0.12	0.211 ± 0.05
15	425	4.41	32.01 ± 1.45	− 43 ± 0.12	0.212 ± 0.06
30	425	4.41	32.02 ± 1.50	− 43 ± 0.12	0.215 ± 0.06
60	425	4.22	33.31 ± 0.39	− 44 ± 0.58	0.241 ± 0.06
120	425	4.03	36.92 ± 1.02	− 45 ± 0.64	0.301 ± 0.07
240	425	3.91	37.51 ± 1.71	− 47 ± 0.81	0.321 ± 0.09

Where the comparison tests were performed for values of similar responses (p < 0.05)

### Antimicrobial activity of Ag-Carth-NPs

#### Inhibition zone, MIC, MBC and Mechanism of action

**Fig. 2:**
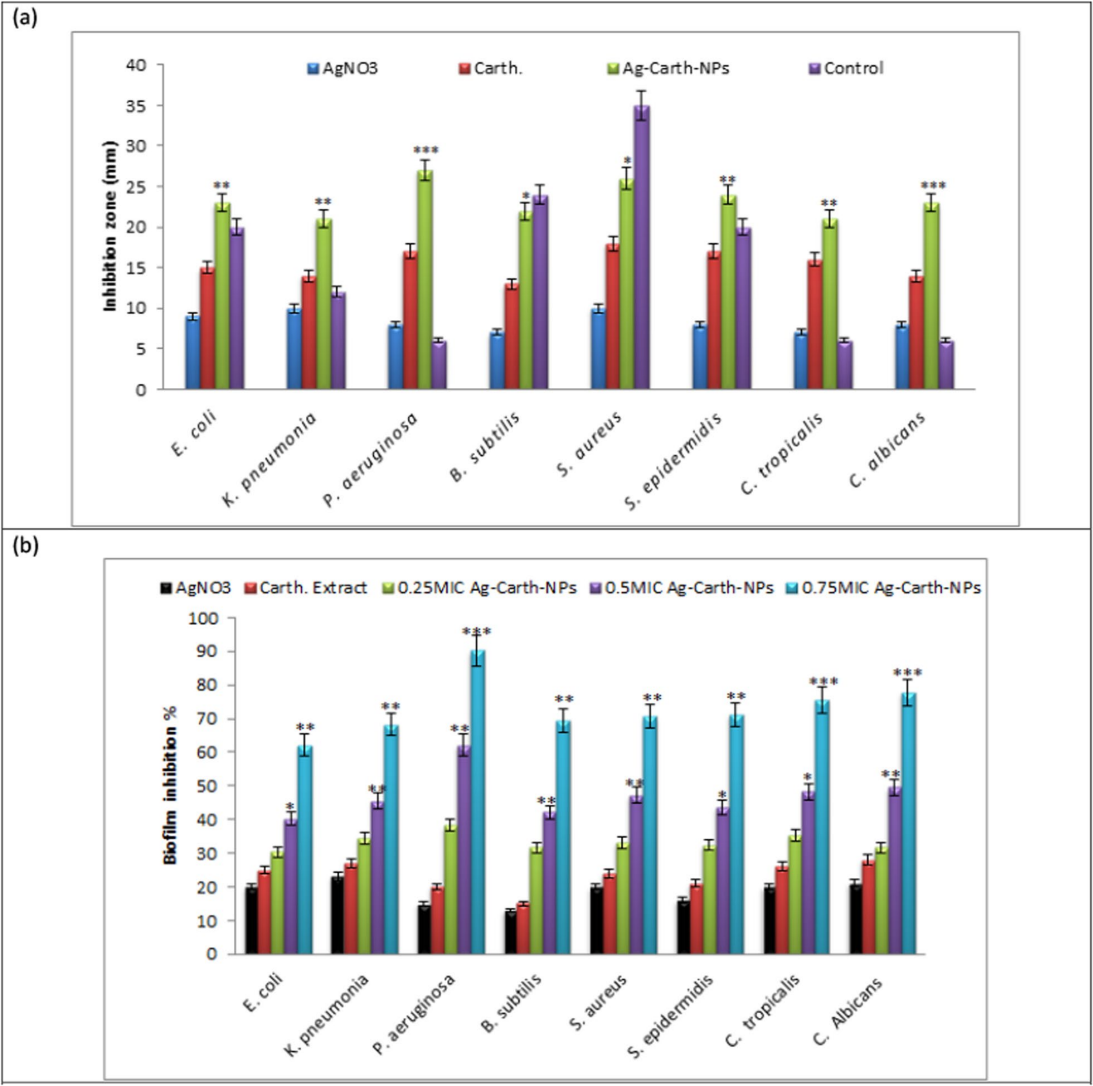
**a** refer to inhibition zone (mm) of AgNO3, *Carthamus* extract, and Ag-Carth-NPs against tested organisms; Where, Clindamycin 2 μg/ml, Nystatin 100 μg/ml as positive control for bacteria and fungi respectively; Experiments were performed in triplicates and **b** refer to Biofilm inhibition % of silver nitrate, *Carthamus* extract and Sub-MIC Ag-Carth-NPs. Mean values with standard deviation (error bars) with *, **, ***are statistically different from the respective control at P < 0.05, P < 0.01, and P < 0.001, respectively; (One way ANOVA, Tukey test)

Natural compounds have distinct antibacterial activities against both gram-positive and gram-negative bacteria [107]. Using biogenic synthesis to create nanoparticles (NPs) through the redox balance of an natural active substance with metal is one method of enhancing antimicrobial action [108]. In this study, the inhibition zone values of AgNO_3_, *Carthamus* extract, and Ag-Carth-NPs were represent in (Figure 2a) in compere with positive control (Clindamycin for bacteria and Nystatin for fungi. The diameter of the inhibition zone demonstrated the antibacterial activity of Ag-Carth-NPs against the examined microorganisms, which was found to be more significant than AgNO_3_, *Carthamus* extract. *P. aeruginosa* is the most common gram-negative pathogen causing multidrug resistant infections [109]. The results showed that the *P. aeruginosa* strain is more resistant to clindamycin antibiotic disc and is also the most susceptible strain to the Ag-Carth-NPs. Inhibition zone values of Ag-Carth-NPs are 23 ± 0.17, 21.0 ± 0.14, 27.0 ± 0.30, 22.0 ± 0.13, 26.0 ± 0.19, 24.0 ± 0.12, 21.0 ± 0.15 and 23.0 ± 0.14 against *E. coli, K. pneumonia, P. aeruginosa, B. subtilis, S. aureus, S. epidermidis, C. tropicalis* and *C. albicans* respectively.

A further investigation in estimating the antibacterial activity was performed by determining the MIC and MBC (Table 3). The MIC of Ag-Carth-NPs was defined as the lowest concentration at which significant inhibition of bacterial growth was achieved. *P. aeruginosa* revealed an MIC value of 3.126 µgml^−1^ for Ag-Carth-NPs, >100 µgml^−1^ for AgNO_3_ and 100 µgml^−1^ for *Carthamus* extract, in that more potent inhibition has been observed in the case of Ag-Carth-NPs against all tested organism. The results show Ag-Carth-NPs has MIC at 6.25 ± 1.05 µgml^−1^ for *E. coli* and *K. pneumonia,* 12.5 ± 1.051 µgml^−1^ for *S. aureus*, *S. epidermidis* and *C. albicans* and 25 ± 1.071 µgml^−1^ for *B. subtilis* and *C. tropicalis*. Previous study demonstrated MIC of myco-synthesized silver nanoparticles for different bacterial strains was 1.5625 µg/ml for *Listeria* and *Shigella*, 0.78125 for *E. coli* and 3.125 for *S. typhi*[110]. The MICof AgNPs against *S. aureus* was 2.5 µg/disc and less than 2.5 µg/disc for *P. aeruginosa*[111]. Other studies refer biogenic AgNPs showed antimicrobial activities against gram-positive and gram-negative bacteria at MIC ranged between 16 and 64 μg ml^−1^[112].

**Table 3:** MIC and MBC of AgNO_3_, *Carthamus* extract, and Ag-Carth-NPs

Isolates strains	MIC (µg/ml)			MBC (µg/ml)
	Ag-NO3	*Carthamus* extract	Ag-Carth-NPs	Ag-Carth-NPs
*E. coli RCMB 0020B01*	> 100	100	6.25^ad^ ± 1.05	12.5^c^ ± 1.061
*K. pneumonia* ATTC 13883	> 100	100	6.25^be^ ± 0.92	12.5^e^ ± 1.041
*P. aeruginosa* clinical isolate	> 100	100	3.126^a^ ± 0.83	6.25^ae^ ± 0.72
*B. subtilis* ATCC 6633	> 100	> 100	25^be^ ± 1.031	50^a^ ± 1.021
*S. aureus* ATCC 25923	> 100	100	12.5^c^ ± 1.051	25^a^ ± 1.71
*S. epidermidis* ATTC 12228	> 100	> 100	12.5^cd^ ± 1.061	25^ad^ ± 1.096
*C. C. tropicalis RCMB 001Y004*	> 100	> 100	25^ae^ ± 1.071	50^d^ ± 1.017
*C. albicans* ATCC 10231	> 100	> 100	12.5^b^ ± 0.925	25^ab^ ± 1.082
LSD	–	–	1.002	1.061

Values are mean ± SD (n = 1.00). Data within the groups are analyzed using one-way analysis of variance (ANOVA)

*LSD* least significant differences

^a, b, c, d, e^Duncan’s multiple range test at p < 0.05

The lowest concentration of NPs that was bactericidal, i.e. that showed no growth on agar plates, was selected as MBC. In this study, for *P. aeruginosa* in case of Ag-Carth-NPs the MBC was 6.25 µgml^−1^. In case of other tested organisms Ag-Carth-NPs showed bactericidal activity at 12.5 for both *E. coli* and *K. pneumonia* and 25 µgml^−1^ for *S. aureus, S. epidermidis* and *C. albicans* and 50 µgml^−1^ for *B. subtilis* and *C. tropicalis.* Previous study refer to biogenic silver nanoparticles has MBC values against *P. aeruginosa *27853 and *S. aureus 25923* at 12 μg ml^−1^ and 3 μg ml^−1^ for *E. coli 35218 *[113]. The biogenic silver nanoparticles were found to be more potent bactericidal agents at low concentration [114].

Previous study indicates that majority of the examined bacteria were not inhibited in growth by aqueous extracts of safflower, with the exception of *Acinetobacter baumannii*, for which the inhibition zone measured two millimeters [115]. The antibacterial properties of silver nanoparticles synthesized from plant extract may have different processes when applied to gram positive and gram negative cells, where production of reactive oxygen species (ROS), radicals OH and hydrogen peroxide, is one of those mechanisms [116]. Especially when it involves gram-negative bacteria, reactive oxygen species cause oxidative stress and target the lipids in the outer membrane, leading to lipid oxidation, damage to proteins, RNA, and DNA, and ultimately, cell death [117]. Fecal microbiota transplantation has been proposed as a potential therapeutic solution [118] Several authors have reported that the microbial activity of AgNPs measuring 20–80 nm was attributed to the release of silver ions [119].

Silver nanoparticles, are recognized as a superior antibacterial agent than other metallic nanoparticles or antimicrobial agents that can fight both in vivo and in vitro microbes that cause diseases [120]. AgNPs have the ability to combat both gram-positive and gram-negative bacteria and fungi, including those that are resistant to multiple drugs [121]. AgNPs have several simultaneous modes of action. They have also demonstrated a synergistic effect against pathogen microbes when combined with antimicrobial agents or antibiotics [122]. Due to their unique properties, silver nanoparticles can be effectively used to cure or prevent infections in a variety of medical and healthcare goods [123]. Advantages of using AgNPs as new antibacterial agents in combination with antibiotic, which will reduce the dosage needed and prevent secondary effects associated to both [124].

*Proposal antimicrobial Mechanisms of Ag-Carth-NPs* Ag-Carth-NPs It is very likely that it has the same mechanism as the AgNPs. Different hypotheses for antimicrobial mechanism of AgNPs are discussed, including (i) Penetrating bacterial membrane, causing the cell membrane to be destroyed and content to spill out [125]. (ii) disrupt DNA structure, or directly contact with DNA to cause DNA mutations and disrupt DNA replication [126], (iii) Generating ROS and disabling the respiratory chains and iv) Inactivating enzymes and denaturing proteins [127]. AgNPs exhibit good antifungal properties against Candida spp. and resistant fungus. For instance, AgNPs may influence drug sensitivities by targeting many cellular targets of *Candida albicans*, such as fatty acids like oleic acid, which are crucial for the hyphal morphogenesis responsible for the pathogenicity [128]. AgNPs may be able to bind to and saturate the fungal hypoha, ultimately rendering the fungus inactive [129]. AgNPs have been proven to effectively inhibit various pathogenic bacteria, fungi and viruses, including gram positive and negative bacteria and fungi and viruses [29, 130]. Some factors can effect on antimicrobial activity of AgNPs such as bacteria strains, size, shape and concentration of AgNPs, time contact, and surface charge of particles [131]. AgNPs with larger surface has larger reaction surface, show stronger antibacterial activity [132]. Microorganism surfaces typically exhibit negative charge [133]. Antimicrobial activity of AgNPs is also affected by the surface charge, so positive charge can facilitate the adherence of Ag-NPs on bacterial membranes through electrostatic attraction [134]. Therefore, adjusting the surface charges of AgNPs may contribute to the enhanced antibacterial effect [135].

Surface interactions between polyphenols and newly reduced metallic silver particles function as the capping factor preventing the agglomeration of AgNPs [136]. Combining silver nanoparticles and plant extracts against microbes has drawn attention within the past few years [137]. Synergistic effect of plant extracts and silver nanoparticles allowed for efficacy higher than that of antibiotics (ampicillin) when tested at the same concentrations and after a relatively short exposure time of 3 h [138]. Preparation of silver nanoparticles in combination with *C. tenuis* extract in form of Ag-Carth-NPs, it is a preferred method and has a strong effect on all tested microbes.

Nanomaterials are used in a variety of biomedical applications, including anticancer therapeutics, antiviral applications, antibacterial and antifungal applications, wound healing, and antitumor activity [139, 140]. Green synthesis methods for silver nanoparticles are currently being developed by researchers and are beneficial for biomedical applications [141, 142]. Because unique characteristics of Ag-NPs such as their higher surface to volume ratio, a wide surface area, strong surface Plasmon resonance, stability, ease of processing, and multi-functionalization are well suited for a variety of biological applications such as antibacterial, antifungal applications, wound healing, target therapy and antitumor activity [133]. Silver nanoparticles, however, have a lot of interesting potential for use in biomedical applications, such as coatings for medical devices, formulations, drug administration, detection and diagnosis platforms, and biomaterials [143].

#### Biofilm inhibition

**Fig. 3:**
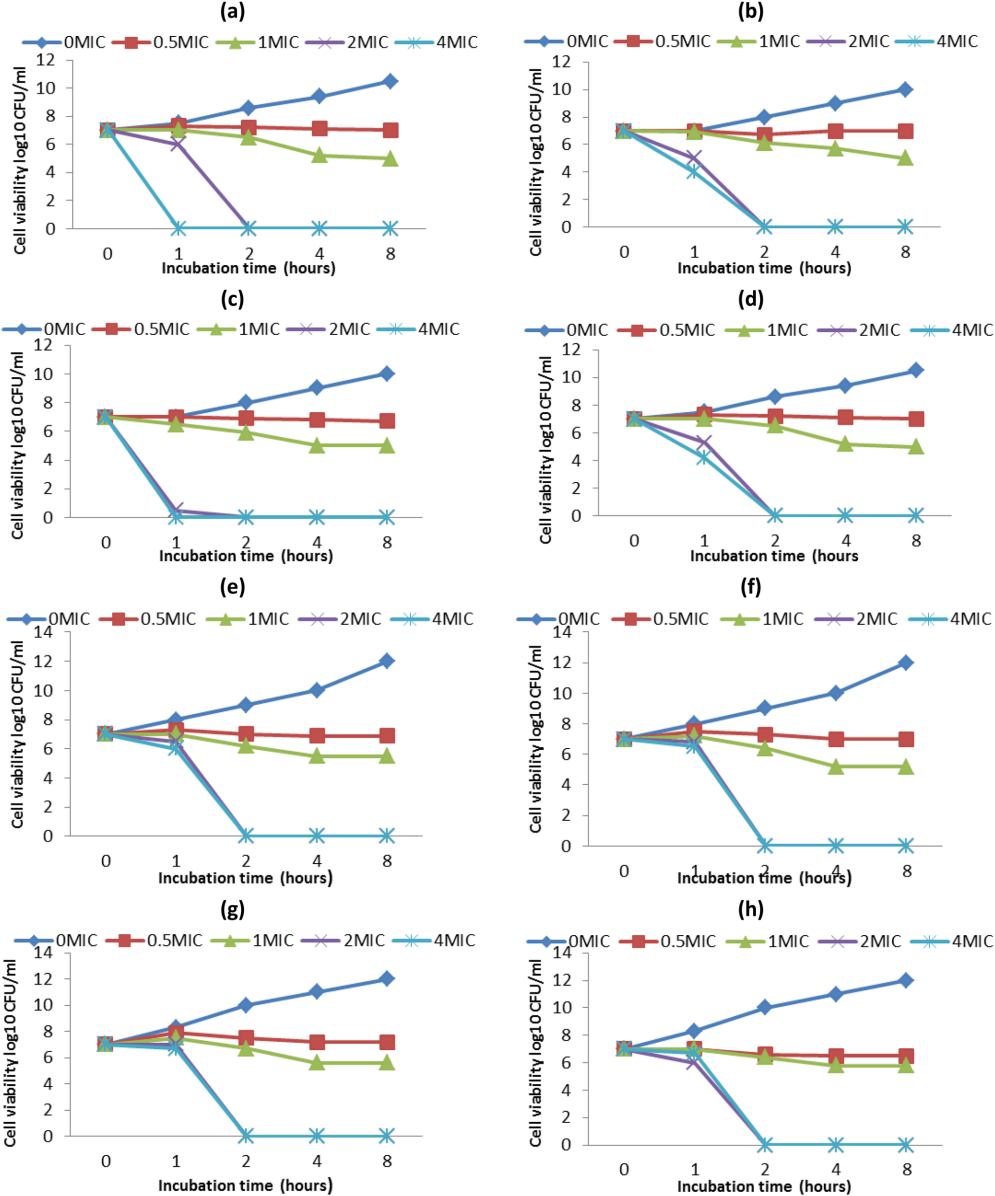
Time kill curve of Ag-Carth-NPs against tested organisms at different concentration and time length. Untreated bacteria/fungi refer to growth at 0MIC and Minimum inhibitory concentration (MIC) is different according to MIC of each microbe. Where; **a** E. coli, **b** K. pneumonia, **c** P. aeruginosa, **d** B. subtilis, **e** S. aureus, **f** S. epidermidis, **g** C. tropicalis and **h** C. albicans. Mean values with standard deviation (error bars) with *, **, *** are statistically different from the respective control at P < 0.05, P < 0.01, and P < 0.001, respectively; (One way ANOVA, Tukey test)

Microbes producing biofilms are the cause of numerous diseases. According to one study by the Centers for Disease Control and the National Institutes of Health, between 65 and 80% of infections were caused by bacteria producing biofilms [144]. The biofilm inhibitory activities of the Ag-Carth-NPs were performed using crystal violet assay. The Ag-Carth-NPs showed significant reduction in the biofilm formation compared to silver nitrate and *Carthamus* extract (positive control) as shown in (Figure 3). It was observed that Ag-Carth-NPs were able to reduce the biofilm formed by *E. col, K. pneumonia, P. aeruginosa, B. subtilis, S. aureus, S. epidermidis, C. tropicalis and C. albicans* are 62.12, 68.25, 90.12, 69.51, 70.61, 71.12, 75.51 and 77.71 %, respectively at 0.75MIC and 40.21, 45.51, 62.12, 42.12, 47.3, 43.61, 48.21 and 49.6% at 0.5MIC and 30.31, 34.5, 38.32, 31.61, 33.21, 32.41, 35.41 and 31.61% at 0.25MIC respectively.

The negative controls is experiment tubes containing media alone represented no growth or biofilm formation in the other hand negative controls is experiment tube contain media plus tested organisms in absence of Ag-Carth-NPs represented microbial biofilm formation without any percent of biofilm formation inhibition. Ag-Carth-NPs have significant inhibition of biofilm formation in compere with AgNO_3_ and *Carthamus* extract. AgNPs (10 µg/ml) synthesized by plant extract of *G. lanceolarium* were used to treat *P. aeruginosa* for 24 hours, resulting in a >99% reduction in biofilm formation[145]. Ag-NPs significantly eradicated mature biofilms developed by *A. baumannii, K. pneumoniae* and *P. aeruginosa* standard strains and clinical isolates[146]. Ag-NPs exhibited reduction in biofilm formation at range 22–79, 29–87, 12–59, 22–63, and 17–81% against *P. aeruginosa, E. coli, C. violaceum, K. pneumoniae, and S. aureus* respectively at sub-MICs ranging from 1/16 × MIC-1/2 × MIC[147]. Previous study reported biogenic synthesized AgNPs resulted in an 89% inhibition of biofilm formation in *S. aureus* and 75% in *E. coli *[145].

Prior research regarding the anti-biofilm effects of biosynthesized AgNPs against *P. aeruginosa* and *S. epidermidis* has been done by S. Kalishwaralal et al. [66]. The synergistic activity of the AgNPs and plant extract to control the biofilm formation by the pathogenic organisms are discussed by previous study [145]. The antibiofilm activity of AgNPs against P*. aeruginosa, S. aureus (MRSA), S. mutans, and C. albicans* was studied extensively and reported the potentiality of AgNPs [148]. Rolim et al. [149] reported the biofilm eradication of AgNPs against *P. aeruginosa* which supports our current results. Three pathways in particular are significant for the antibiotic resistance of bacteria in biofilms are (i) Resistance at the Biofilm Surface, (ii) Resistance in Biofilm Microenvironments and (3) Persister bacterial cells resistance [150].

Biofilm formation inhibition by Ag-NPs is due to, structural alterations of the membrane, increasing permeability and damaging membrane integrity. Furthermore, Ag-NPs adhered to the cell surface can stop the production of biofilms since bacterial adherence to any surface is the first step in the process [151]. Since Ag ions which generated from Ag-NPs after transported into the cell may interfere with microbial proteins and enzymes which are required for microbial adherences or formation of quorum sensing that resulted in the reduction in biofilm formation [152]. Physical and chemical properties of AgNPs, such as size and shape influence the activity of the nanoparticles, because smaller sizes increase the surface contact area of AgNPs with microorganisms and then decrease biofilm formation [37].

#### Time kill curve activity of Ag-Carth-NPs

The time kill activity of tested pathogens is done at Ag-Carth-NPs final concentration of 0 MIC, 0.5 MIC, 1 MIC, 2MIC and 4MIC and different time interval 0, 1, 2, 4 and 8 h as shown in (Figure 3). Ag-Carth-NPs were effective in inhibiting and killing the microbes in a dose and time dependent manner as shown in the time kill assays. The bactericidal activity of Ag-Carth-NPs is effective against the selected bacteria and fungi; the reduction in the number of CFU/ml is significant in compered with growth of controls (untreated bacteria/fungi refer to growth at 0MIC) that has ascending growth curve over all tested time.

The bactericidal endpoint of Ag-Carth-NPs for *E. coli* was reached after 1 h of incubation at 4MIC (25 µg/ml); while for *K. pneumoniae*, the bacteria was killed after 2 h of incubation at 2MIC (12.5 µg/ml) and after 1 h at 4MIC (25µg/ml). *P. aeruginosa* was killed after 1 h of incubation at 2MIC (6.25 µg/ml) and 4MIC (12.5 µg/ml). The bactericidal endpoint of Ag-Carth-NPs for *B. subtilis* was reached after 2 h of incubation at 2MIC (50 µg/ml) and 4MIC (100 µg/ml); however, the end point reached after 2h of incubation at 2MIC (25 µg/ml) and (100 µg/ml) for *S. aureus*. The bactericidal endpoint of Ag-Carth-NPs for *S. epidermidis* was reached after 2 h of incubation at 2 MIC (25 µg/ml) and 4MIC (50 µg/ml). *C. tropicalis* was killed after 2 h of incubation at 2MIC (50 µg/ml) and 4MIC (100 µg/ml); however, the bactericidal endpoint of Ag-Carth-NPs for *C. albicans* after 2 h of incubation at 2 MIC (25 µg/ml) and 4MIC (50 µg/ml).

The results demonstrated that Ag-Carth-NPs could completely inhibit growth of the tested microorganisms in dose dependent manner. A Significant difference was found among the tested pathogens at the time killing depend on types of organisms and concentration of Ag-Carth-NPs. However, the end point reached faster after 1h of incubation with low concentration for *P. aeruginosa* in competing with other tested microbes. Ag-NP concentrations and the types of bacteria used in the research have an impact on the inhibition of bacterial growth [153]. Ag-Carth-NPs exhibit significant bactericidal effect against all tested organisms, this must be due to synergistic effect of AgNPs and bioactive agent of *Carthamus* extract present over its surface as reported earlier with AgNPs plant extract combination [154]. Furthermore, such surface loading biochemical agents over NPs enhance solubility of NPs in aqueous suspension, thereby providing the additional benefit of increased bioavailability and therapeutic potential [155].

The killing activity of AgNPs are fast acting against all the gram negative bacteria and the reduction in the number of CFU mL-1 was >3 Log10 units (99.9%) in 1–2 h [9]. The bactericidal ability of Ag-NPs synthesized by *S*. *polyanthum* leaves extract entirely killed most foodborne pathogens after 4 h of incubation at 4 × MIC concentration [156]. AgNPs reduced the number of the bacterial cells by more than 3 log10 when compared to the initial inoculum [157]. Based on the results, the tested gram negative bacteria were able to kill in a shorter time at low concentration of Ag-Carth-NPs compere with positive bacteria and fungi. This may be attributed to the composition of bacteria's cell walls. Compared to gram positive bacteria, gram negative bacteria have a distinct cell wall structure, an outer membrane made of lipopolysaccharide, a thin layer of peptidoglycan, and a cytoplasmic membrane [158]. Due to their potent biocidal impact against pathogens, silver nanoparticles are widely recognized as among the most common antimicrobial agents [159] and AgNPs have been used as anti-fungal [160]. The smaller NPs have a higher surface area than bigger ones; they may be more bactericidal to bacteria and exhibit superior bactericidal effects because they can more easily attach to the cell wall and penetrate the bacterial cell [161].

#### Motility of *P. aeruginosa*

**Fig. 4:**
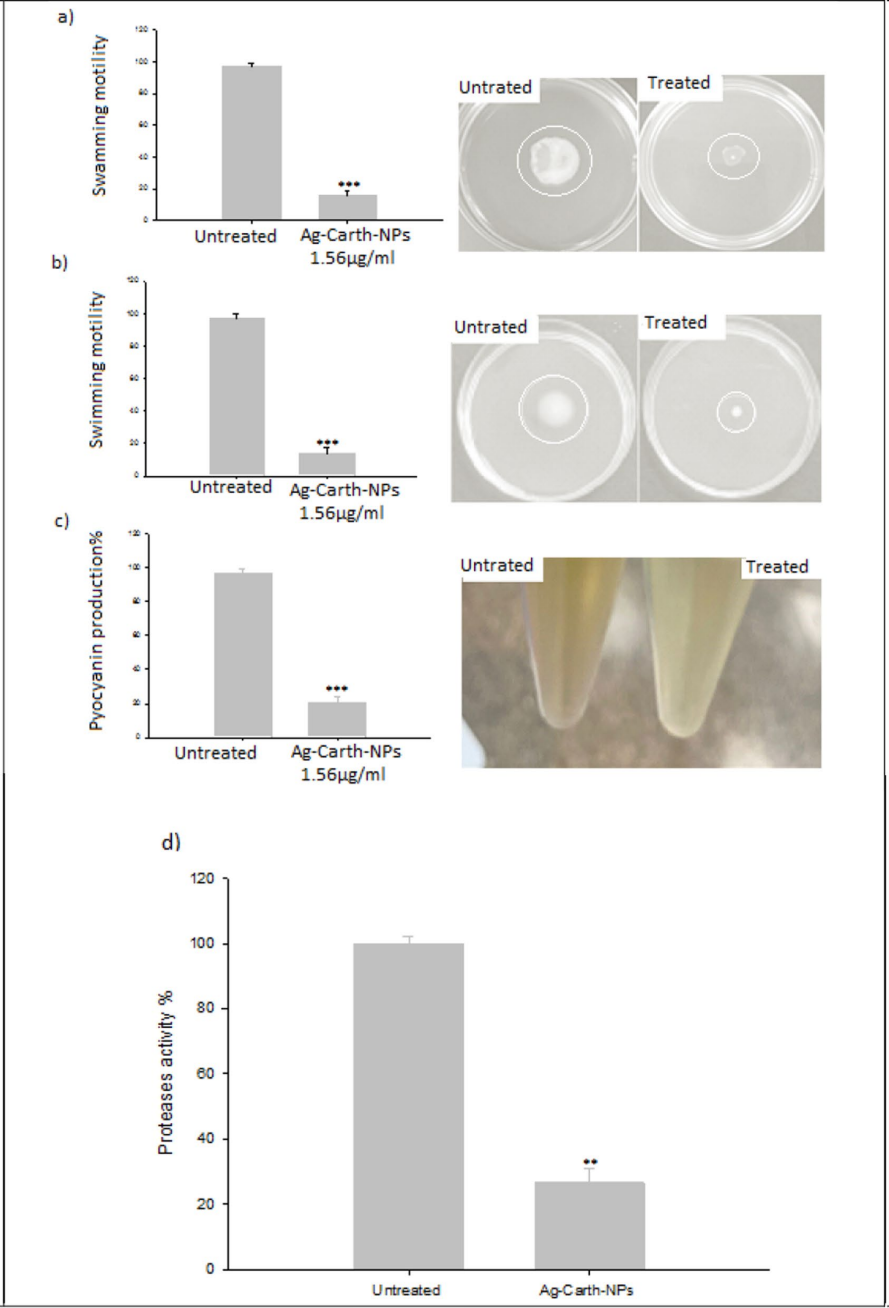
**a** Swarming motility inhibition of *P. aeruginosa* by sub-MICs of Ag-Carth-NPs, **b** Swimming motility inhibition of *P. aeruginosa* by sub-MICs of Ag-Carth-NPs, **c** Pyocyanin production inhibition of *P. aeruginosa* by sub-MICs of Ag-Carth-NPs and **d** Proteases production inhibition of *P. aeruginosa* by sub-MICs of Ag-Carth-NPs. Mean values with standard deviation (error bars) with *, **, ***are statistically different from the respective control at P < 0.05, P < 0.01, and P < 0.001, respectively; (One way ANOVA, Tukey test)

The sub-MIC inhibitory effect of Ag-Carth-NPs on different types of virulence factors in *P. aeruginosa* was investigated (Selected because is more resistant strain to control antibiotic and high sensitive to Ag-Carth-NPs). The Ag-Carth-NPs had a significant influence on the swarming, motility of *P. aeruginosa* when they were present at concentrations of 1.56 μg/ml as compared to the untreated controls as shown in (Figure 4a). Ag-Carth-NPs show maximum inhibition of swarming motility about 84.23% in compere with control (untreated samples). Since *P. aeruginosa* motility, which includes properties like swarming, promotes more biofilm development and the surface attachment process [162]. In contrast Ag-Carth-NPs showed a remarkable reduction in bacterial swimming (by 86.59%) after treatment with Ag-Carth-NPs (Figure 4b).

One potential strategy for managing biofilm and infections is to use NPs to reduce microbial motility [163]. Ag-Carth-NPs effectively reduce swarming motility at sub-MIC levels, which is similar with the previous NPs inhibitory effects when made from other natural products [164]. The biosynthesized Ag-NPs have been observed to decrease the swarming motility and biofilm formation in *P. aeruginosa *[147]. Silver metformin nanostructure show swarming motility inhibition by about (88.87–94.16%) [50]. The previous study demonstrated that AgNPs show 42–81 % reduction in motility behavior of *P. aeruginosa *[145]. It has also been shown that the sub-MIC level of Ag-Carth-NPs inhibits the formation of biofilm in *P. aeruginosa*, as well as inhibits the swarming motility activity.

#### Pyocyanin level

Treating of *P. aeruginosa* with Ag-Carth-NPs reduced by 78.71% (Figure 4c); Based on results of ANOVA, it was found that, the of OD value of pyocyanin produced by *P. aeruginosa* was significantly reduced compared to the control (untreated sample). Treatment of *Pseudomonas aeruginosa* with the Ag-NPs (0.5–1 μg/mL) resulted in a significant a significant decrease of production of pyocyanin [165]. In a study, Khan et al, showed that the production of *P. aeruginosa* PAO1 KCTC 1637 pyocyanin at gold nanoparticles concentrations of 0.032, 0.128 and 0.256 mg/ml decreased 79.4, 81.9, and 87.7%, respectively compared to the control [164]. Comparable to the findings of this investigation, demonstrated the impact of nanoparticles on decreasing *P. aeruginosa* pyocyanin production [166]. Thus, without influencing bacterial growth or starting resistance selection, pyocyanin decrease can be thought of as an effective way to reduce the pathogenicity and colonization of P. aeruginosa [167].

#### Total protease production 

The ability of the Ag-Carth-NPs to inhibit proteolytic activity was measured using the modified skimmed milk broth method. It was found that the inhibitory activity of Ag-Carth-NPs is 73.8% in compere with control (Figure 4d). Proteases destroy immune globulins and fibrin as well as they disrupts epithelial tight junctions [168]. Nanoparticles has significant capability to inhibit *P. aerouginosa* Proteases production [169]. Significantly reduced the levels of total protease by biologically Synthesized gold and selenium Nanoparticles [48].

#### Virulence attenuating properties of Ag-Carth-NPs against *P.aeruginosa*

##### Conventional PCR for virulence genes screening

**Fig. 5:**
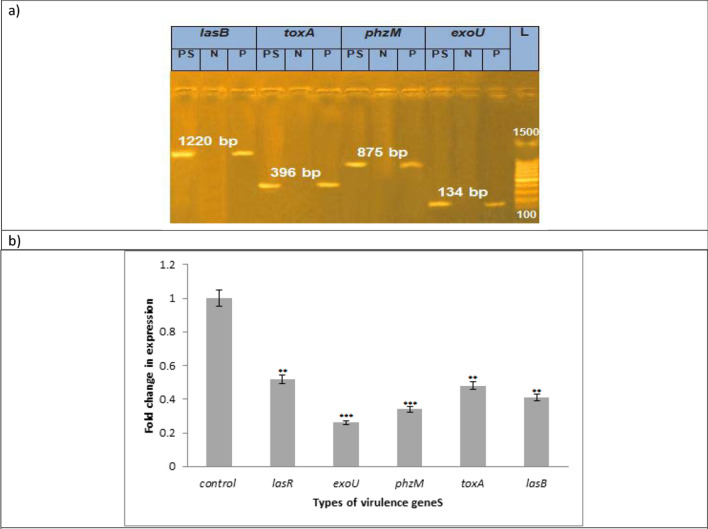
**a** Screening of the major *P. aerugenosae* virulence genes and **b** RT-qPCR showed reduced expression of *lasR, exoU, phzM, toxA* and *lasB* with the Ag-Carth-NPs in sub-MICs compared to untreated controls. Mean values with standard deviation (error bars) with *, **, ***are statistically different from the respective control at P < 0.05, P < 0.01, and P < 0.001, respectively; (One way ANOVA, Tukey test)

Upon screening of the major *P. aerugenosae* virulence genes, the isolate was positive for *exoU, phzM, toxA* and *lasB* showing bands at 134, 875, 396, and 1220 bp, respectively. Therefore, this clinical isolate can be identified as a hypervirulent strain (Figure 5a). Previous study confirm virulence genes, of *ExoU, ExoS, phzM, toxA*, and *lasB* genes are present in *P. aeruginosa* isolate [170]. The presence of different virulence genes in *P. aeruginosa* isolates indicates that these strains have a relationship to various levels of intrinsic pathogenicity and virulence[171]. The *phz, lasB, toxA, exoS and exoU* genes were detected in *P. aeruginosa* strains exhibit high antimicrobial resistance [172].

##### Assessment of the effect of of Ag-Carth-NPs genes using qRT‑PCR

The DNA extract were tested for the presence of the five exotoxin genes (*lasR, exoU, phzM, toxA* and *lasB*) in *P. aeruginosa* using specific primers by multiplex PCR. A variety of virulence factors play a role in the pathogenesis of *P. aeruginosa* [45]. The inhibition of the expression of *P. aeruginosa* virulence genes (*lasR, exoU, phzM, toxA* and *lasB*) were assessed by qRT-PCR after 24 h of treatment with 1.562 µg/ml Ag-Carth-NPs. The expression levels of *lasR, exoU, phzM, toxA* and *lasB* were significantly decreased after treatment with sub-MICs of the Ag-Carth-NPs compared to controls (Figure 5b). The results show, *ToxA* gene expression was significantly downregulated by 81.5%, while *exoU* gene expression reduced by 78.1%. The percentage inhibition in *lasR* gene expression was 68%, while the reduction in *exoU* was 66%. Furthermore, there was a 60.1% decrease in *lasB* gene expression.

Biogenic synthesized Ag-NP show a significant reduction in *LasB* production by *P. aeruginosa* [173]. The expression of QS regulatory genes (*lasI, lasR, rhlI, rhlR, and fabH2*) in *P. aeruginosa PAO1* was suppressed by AgNPs, according to Singh et al, [174]. The combination of AgNPs and 4-nitropyridine N-oxide can inhibit the expression genes (*lasI, lasR, rhlI, rhlR, pqsA, and pqsR*) [175]. A significant reduction in virulence gene expression was observed with chemical synthesized AgNPs [50, 176]. Previous study refer to Ag-CNTs, the expression levels of the *rpoS, rsmZ*, and *oprD* genes were significantly down regulated in *P. aeruginosa* compared to the untreated samples [51]. These results suggest that the mechanism of action may be attributed to their effect on cell-membrane integrity, down regulation of virulence-gene expression, and induction of general and oxidative stress in *P. aeruginosa*

Ag nanoparticles (NPs), exhibits superior ion release per unit mass, essentially owing to the augmented effective surface area. The dynamic correlation between nanoscale effects and Ag(I) ions contributes to the enhanced antimicrobial efficacy of Ag-NPs compared to their bulk counterparts [177]. It raises three probable antimicrobial mechanisms in Ag-NPs, including Ag-NPs as a reservoir for Ag(I) ions, particle only effects, and synergistic effects due to the combination of the first two mechanisms [178]. Synergistic antimicrobial mechanism, in which the NPs are absorbed intracellular and endure subsequent leaching of Ag+, raising local ion concentrations, resulting in physical interference or disruption of the phospholipid cell membrane and ROS generation at the surface of cell biomolecules [179]. This leads to damage the biomolecules such as enzymes, proteins and DNA, resulting in bacterial cell death [180].

#### Possible strategies for Ag-Carth-NPs from the environment

There is extensive research on the application of Nano based materials and the consequences of their release into the environment. However, there is little information about environmentally friendly approaches for removing nanomaterial’s from the environment [181]. Nanomaterial’s especially AgNPs affect soil properties, microorganisms, and plants and can therefore cause toxicity for living organisms including humans. However, there is little information about environmentally friendly approaches for removing nanomaterials from the environment. The possibility of remediation of Ag-Carth-NPs using phytotechnology approaches. Phytotechnology refers to a technology that uses plants to remove, uptake, absorb, transform, transfer, attenuate, accumulate, degrade, or metabolite organic, inorganic, metallic, or metalloid contaminants from soil, water, or air [182]. The term phytotechnology refers to a group of sub-disciplines of scientific study that include phytoextraction, phytosequestration, phytovolatilization, phytodegradation, and phytoremediation. The terms phytotechnology and phytoremediation are synonymous, however the latter term usually designates a phytotechnological process that eliminates contaminants from the surrounding environment. By storing contaminants in plant biomass to assure environmental safety, phytoremediation is a natural, easy, affordable, and widely used bioremediation approach that uses the principles of a plant’s metabolic system to clean, recover, and remediate contaminated environments [183].

#### Cytotoxicity of Ag-Carth-NPs

**Fig. 6:**
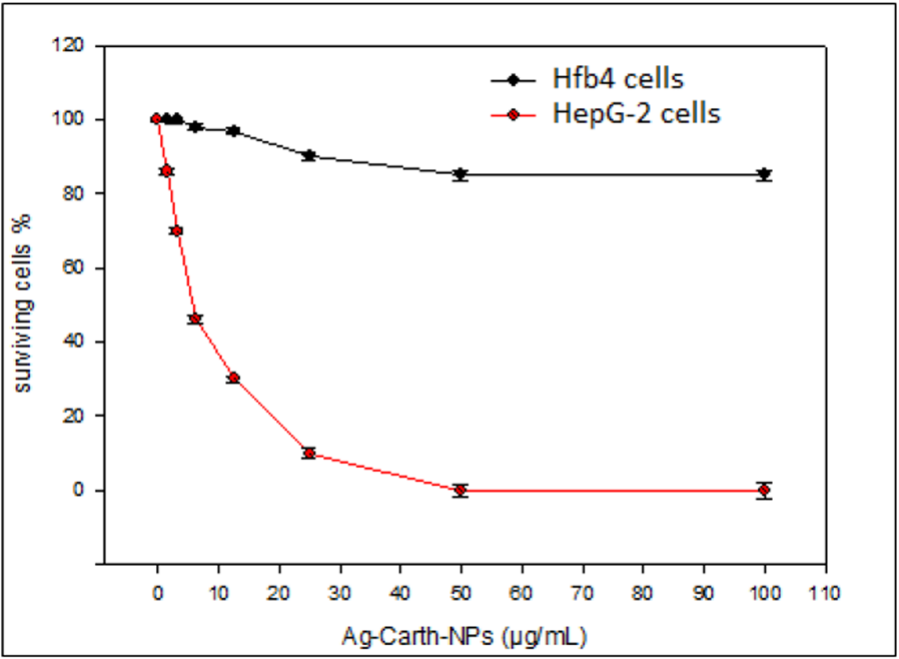
Cytotoxicity of Ag-Carth-NPs against Hfb4 cells (normal skin cell lines) and HepG-2 cells (human Hepatocellular carcinoma). Ag-Carth-NPs have no cytotoxic effects on the normal tested cell lines. IC50 value of Ag-Carth-NPs against HepG-2 cell lines is 5.6 µg/mL. Mean values with standard deviation (error bars) with *, **, ***are statistically different from the respective control at P < 0.05, P < 0.01, and P < 0.001, respectively; (One way ANOVA, Tukey test)

The potential application of Ag-Carth-NPs as antimicrobial agent must be taken into account that it is not harmful to the normal. The results in this, Ag-Carth-NPs have no significant cytotoxic effects on normal human cell (Hfb4) at different concentration ranged from 100 to 1.56 (Figure 6) this indicates Ag-Carth-NPs are safe against human normal cells. The anticancer efficacy of Ag-Carth-NPs has been investigated against HepG-2 cells (human Hepatocellular carcinoma). As the concentrations of Ag-Carth-NPs increased, cell viability decreased, meaning cytotoxicity increased (Figure 6). Data analysis confirms that the IC_50_ value of Ag-Carth-NPs against HepG-2 cells is 5.6 µg/ml.

Ag-NP exposure may cause morphological abnormalities in cells, decrease viability in cells, increase the release of lactate dehydrogenase (LDH), and ultimately cause necrosis and apoptosis in cells. The direct result of AgNP-induced oxidative stress and Ag ion release is cytotoxicity [184]. Biologically synthesized Ag-NP were also proved to exhibit excellent cytotoxic effect on MCF-7 and HT-29 [185]. Human body can tolerate 0.4 to 27 μg of AgNPs per day when consumed through the oral route [186]. Human cells were found to have a greater resistance to the toxic effects of silver nanoparticles in comparison with other cells [187]. Green synthesized AgNPs are biocompatible and do not harm normally functioning human or host cells [188].

## Conclusion

In the present study, a facile biological technology is used for Ag-Carth-NPs synthesis in the presence of Silver nitrate and *Carthamus tenuis* extract. The synthesized Ag-Carth-NPs demonstrated spherical particles with 20  ±  1.22 nm, high stability with zeta potential around – 43 mV and FTIR spectroscopy indicated the presence of various functional biological groups that responsible for stabilization of Ag-Carth-NPs. Antimicrobial results revealed that Ag-Carth-NPs has inhibition zone ranged (27 ± 0.30 to 21 ± 0.14 mm) and anti-biofilm formation activities ranged (40.21 to 90.12%) against tested bacteria and fungi. The values of MIC and MBC ranged between 3.126 and 25 and 6.25–50 μg/ml, respectively. Ag-Carth-NPs inhibit the growth of the tested microorganisms at 2MIC after times ranging from 1 h to 2 h. Furthermore Ag-Carth-NPs reduce swarming, swimming motility, pyocyanin and protease production of *P. aeruginosa* in comparison with the control (untreated). The results show, *P. aeruginosa ToxA* gene expression was significantly down regulated by 81.5%, while *exoU* gene expression was reduced by 78.1%. The percentage inhibition in *lasR* gene expression was 68%, while the reduction in *exoU* was 66%. Furthermore, there was a 60.1% decrease in *lasB* gene expression. Ag-Carth-NPs have no significant cytotoxic effects on normal human cell (Hfb4) but have IC_50_ at 5.6 µg/ml against of HepG-2 cells. Further carefully designed studies should be taken taking into account such as, pharmacokinetic, and formulation of gel based Ag-Carth-NPs are necessary to demonstrate the possibility usage as a promising novel drug for combating pathogenic microbes in topical form.

## Supplementary Information

Additional file 1.

## Data Availability

The data and materials that support the findings of this study are available from the corresponding author, upon reasonable request. No datasets were generated or analysed during the current study.
