# Supplementary material for: Impeding microbial biofilm formation and Pseudomonas aeruginosa virulence genes using biologically synthesized silver Carthamus nanoparticles
Source: Microb Cell Fact. 2024 Sep 5;23:240. doi: 10.1186/s12934-024-02508-9 (PMC11378559; doi:10.1186/s12934-024-02508-9)
Supplement: Supplementary file 1 — Additional file 1. [file 12934_2024_2508_MOESM1_ESM.docx]

**Supplementary data**

Preparation and sterilizing the agar plates was done according to previous described study [57], with slightly significant modification **(Supplementary data).**

**Operating procedure to make 1 liter media:**

1. Using a digital balance, we prepared the medium according to the manufacturer’s instructions. The pH was adjusted to neutral (6.8) at 25°C

2. We placed water in a 1L beaker. Using the hotplate stirrer and magnetic stirring bar, we mixed ingredients with the water whilst stirring to prevent clumping. Heat whilst stirring until the agar dissolves at 95^o^C. The agar was poured into conical flasks and plugged with nonabsorbent cotton wool

3. We sterilize the conical flask using an autoclave 15-20 minutes at 121^o^C, 15psi, according to manufacturer’s instructions for use of this equipment.

4. The pressure in the vessel should be at zero prior to opening the equipment. The sterilized agar is allowed to cool to 50^o^C this is the optimal temperature for pouring to minimize condensation. Agar solidifies at 42^o^C.

5. We thoroughly washed and dried hands, wear a disposable apron.

6. We create a sterile area by thoroughly wiping the bench with 70% alcohol and paper towel.

7. We used the hand holding cotton plug to lift the lid of the Petri dish. Pour approximately 15 to 20mL of the sterilized nutrient agar medium into the base of the Petri dish; until it is about half full, we hold the Petri dish lid so that it partially covers the bottom of the dish as we pour. This prevents microbes and air borne dust particles from dropping into the sterile plate and contaminating it.

10. Immediately we placed the lid on the base at an angle so that steam is able to escape.

11. We poured the remaining plates the same way.

**Table 1s:** Primers sequences, target genes, amplicon sizes and cycling conditions for conventional PC:

| **Target bacteria** | **Target**  **genes** | **Primers sequences** | **Amplified**  **Segment**  **(bp)** | **Primary**  **denaturation** | **Amplification (35 cycles)** | | | **Final extension** | **Reference** |
| --- | --- | --- | --- | --- | --- | --- | --- | --- | --- |
|  |  |  |  |  | **Secondary denaturation** | **Annealing** | **Extension** |  |  |
| ***P.***  ***aeruginosa*** | *exoU* | CCGTTGTGGTGCCGTTGAAG | 134 | 94˚C  5 min. | 94˚C  30 sec. | 55˚C  30 sec. | 72˚C  30 sec. | 72˚C  7 min. | **[72]** |
|  |  | CCAGATGTTCACCGACTCGC |  |  |  |  |  |  |  |
|  | *phzM* | ATGGAGAGCGGGATCGACAG | 875 | 94˚C  5 min. | 94˚C  30 sec. | 54˚C  40 sec. | 72˚C  50 sec. | 72˚C  10 min. | **[73]** |
|  |  | ATGCGGGTTTCCATCGGCAG |  |  |  |  |  |  |  |
|  | *toxA* | GACAACGCCCTCAGCATCACCAGC | 396 | 94˚C  5 min. | 94˚C  30 sec. | 55˚C  40 sec. | 72˚C  40 sec. | 72˚C  10 min. | **[74]** |
|  |  | CGCTGGCCCATTCGCTCCAGCGCT |  |  |  |  |  |  |  |
|  | *lasB* | ACAGGTAGAACGCACGGTTG | 1220 | 94˚C  5 min. | 94˚C  30 sec. | 57˚C  40 sec. | 72˚C  1.2 min | 72˚C  12 min. | **[73]** |
|  |  | GATCGACGTGTCCAAACTCC |  |  |  |  |  |  |  |
|  |  | ACCTGGAGGAGCAGTGAAAG |  |  |  |  |  |  |  |
|  | *Plb1* | ATGATTTTGCATCATTTG | 751 | 94˚C  5 min. | 94˚C  40 sec. | 50˚C  40 sec. | 72˚C  45 sec. | 72˚C  10 min. | **[75]** |
|  |  | AGTATCTGGAGCTCTACC |  |  |  |  |  |  |  |

Where; *exo*U: encoding an important cytotoxin, *phz*M: encoding Pyocyanin, *tox*A: encoding for exotoxin A; *las*B: encoding for lastase B.

**Table 2s:** Primers sequences, target genes and cycling conditions for SYBR green rt-PCR

| **Target bacteria** | **Target genes** | **Primers sequences** | **Reverse transcription** | **Primary**  **denaturation** | **Amplification (40 cycles)** | | | **Dissociation curve**  **(1 cycle)** | | | **Reference** |
| --- | --- | --- | --- | --- | --- | --- | --- | --- | --- | --- | --- |
|  |  |  |  |  | **Secondary**  **denaturation** | **Annealing**  **(Optics on)** | **Extension** | **Secondary**  **denaturation** | **Annealing** | **Final denaturation** |  |
| ***P. aeruginosa*** | ***16S rDNA*** | GGGGGATCTTCGGACCTCA | 50˚C  30 min. | 94˚C  15 min. | 94˚C  15 sec. | 52˚C  30 sec. | 72˚C  30 sec. | 94˚C  1 min. | 52˚C  1 min. | 94˚C  1 min. | **[73]** |
|  |  | TCCTTAGAGTGCCCACCCG |  |  |  |  |  |  |  |  |  |
|  | ***exoU*** | CCGTTGTGGTGCCGTTGAAG |  |  |  | 55˚C  30 sec. |  |  | 55˚C  1 min. |  | **[72]** |
|  |  | CCAGATGTTCACCGACTCGC |  |  |  |  |  |  |  |  |  |
|  | ***toxA*** | GACAACGCCCTCAGCATCACCAGC |  |  |  | 55˚C  30 sec. |  |  | 55˚C  1 min. |  | **[75]** |
|  |  | CGCTGGCCCATTCGCTCCAGCGCT |  |  |  |  |  |  |  |  |  |
|  |  | GCT TAC TTT CTA ACA CTA ACG CGC |  |  |  |  |  |  |  |  |  |
